# Relative density clouds: Visualizing and exploring multivariate patterns of group differences

**DOI:** 10.1371/journal.pone.0287784

**Published:** 2023-06-27

**Authors:** Marco Del Giudice

**Affiliations:** Department of Psychology, University of New Mexico, Albuquerque, New Mexico, United States of America; TU Wien: Technische Universitat Wien, AUSTRIA

## Abstract

This paper introduces *relative density clouds*, a simple but powerful method to visualize the relative density of two groups in multivariate space. Relative density clouds employ *k*-nearest neighbor density estimates to provide information about group differences throughout the entire distribution of the variables. The method can also be used to decompose overall group differences into the specific contributions of differences in location, scale, and covariation. Existing relative distribution methods offer a flexible toolkit for the analysis of univariate differences; relative density clouds bring some of the same advantages to fruition in the context of multivariate research. They can assist in the exploration of complex patterns of group differences, and help break them down into simpler, more interpretable effects. An easy-to-use R function is provided to make this visualization method widely accessible to researchers.

## Introduction

Comparisons between groups—broadly conceived to include demographic categories, individuals sampled at different times and locations, allocations to treatment vs. control conditions, or even biological species—arise all the time across scientific disciplines. Traditional approaches to group comparisons focus on differences in central tendency (usually the mean) and, less often, dispersion (typically the variance or derived indices, such as the coefficient of variation). Important as they are, these summary indices only convey limited information about the shape of the distributions. Differences in central tendency, dispersion, and other aspects of the distributions (e.g., skewness) often combine to yield complex patterns of group differences, with different groups being over- or under-represented at different locations. For example, researchers are sometimes interested in directly comparing the representation of two groups at the distribution tails, which can be done with specialized indices such as *tail ratios* [1, 2] or the newly proposed *S-index* [3]. As distributions grow in complexity (with features such as multiple peaks, thick tails, etc.), the resulting patterns of differences may become so intricate that they defy any attempt to describe them using summary indices.

**Fig 1:**
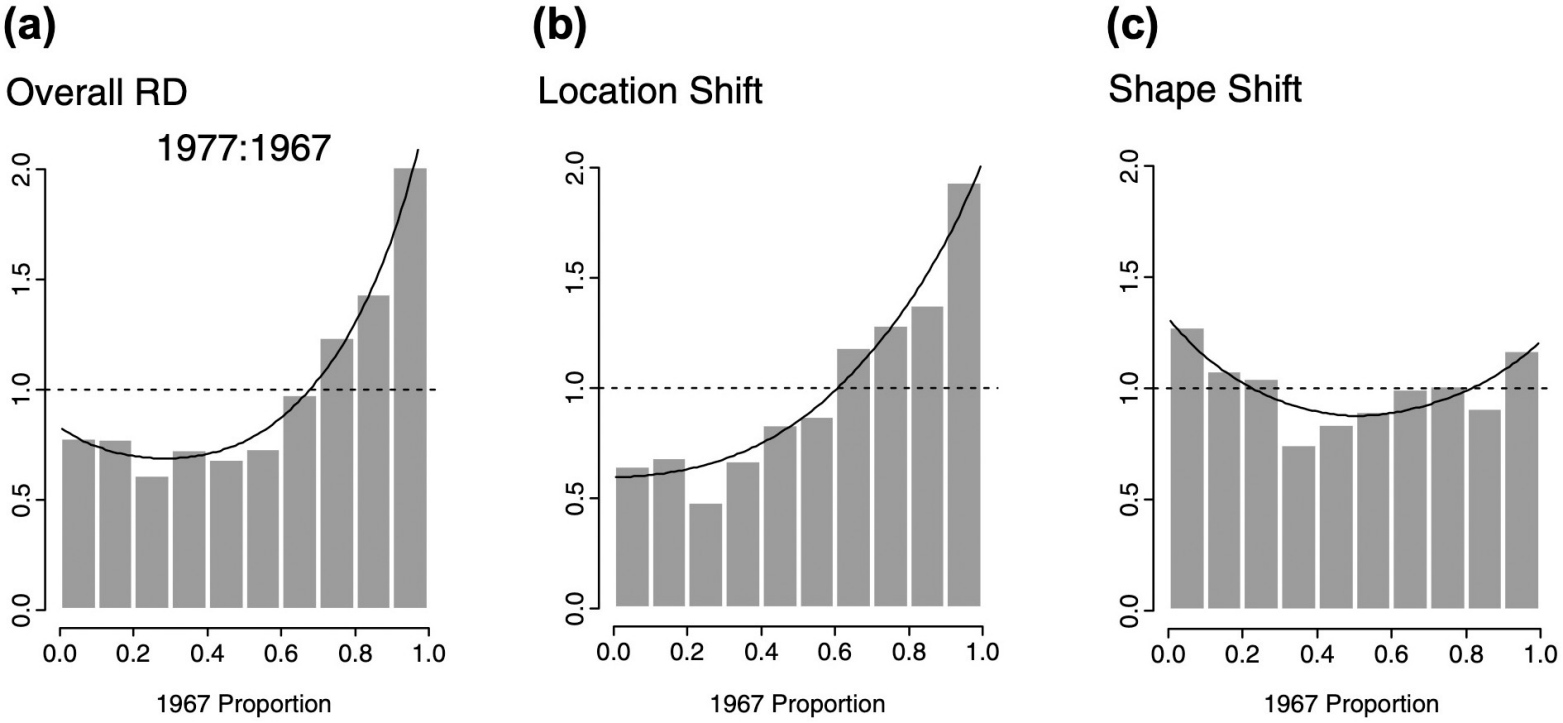
Examples of univariate relative density plots. The plots compare the distribution of white men’s earnings in 1977 to those in 1967 (data from the annual U.S. Current Population Survey). Panel (a) plots the overall relative density (RD) against the quantiles of the 1967 distribution. Panels (b) and (c) decompose the overall difference into the specific effects of location and shape shifts. Reproduced with modifications from [5].

The general task is to devise ways to effectively compare the shape of two distributions across the entire span of the relevant variables, and (if possible) identify the specific contributions of differences in means, variances, and other features to the overall pattern. In univariate contexts, relative distribution methods [4–6] offer a sophisticated toolkit for the analysis of group differences. In particular, *relative density plots* such as those in Fig 1 display the ratio of the densities of two distributions (a comparison group and a reference group) against the quantiles of the reference group. Importantly, when the two groups are approximately equally numerous (e.g., males and females), the relative density directly corresponds to the relative frequency of their members across the range of the target variable. (See [7] for a review of conceptually similar methods based on quantile differences instead of relative densities).

Relative distribution methods can be used to perform various kinds of statistical inference, and to decompose the overall pattern of differences (Fig 1a) into a *location component* that reflects the effect of shifts in location (e.g., differences in means; Fig 1b), and a *shape component* that captures additional differences in dispersion, skewness, and other aspects of the distribution (Fig 1c). The shape component may be further decomposed into a *scale component* (due to e.g., differences in variances) and a *residual shape component* adjusted for both location and scale. In principle, this approach can be extended to yield finer-grained decompositions involving higher moments of the distributions (see Hancock & Morris, 1998, 1999).

To date, relative distribution methods are limited to comparing groups one variable at a time. However, multivariate comparisons that consider several variables at once (as well as their patterns of covariation) can be quite informative in many scientific contexts. Drawing from my own interests, researchers who study psychological sex differences are increasingly tackling multivariate questions—for example about the overall distance between the sexes in multivariate space, or the proportion of overlap between the male and female distributions (e.g., [8–10]; see [1]). Even in these applications, researchers tend to focus mainly on differences between multivariate means or *centroids* (even though tail ratios and variance ratios can be easily generalized to the multivariate case; see [1]). The logical next step is to move beyond means, and begin to investigate complex patterns of differences in the shape of multivariate distributions.

### Relative density clouds

In this paper, I make a contribution in this direction. I introduce a simple yet powerful method to visualize the relative density of two groups in multivariate space, and explore the specific contributions of differences in location, scale, and covariation among variables. The procedure consists of three steps: (1) sample the multivariate distribution at a large number of locations, or “probes”; (2) use a *k*-nearest neighbor (*k-*NN) method to estimate the relative density at each probe; and (3) plot the probes in paired bivariate scatterplots, using color and transparency to visualize local patterns of relative density.

**Fig 2:**
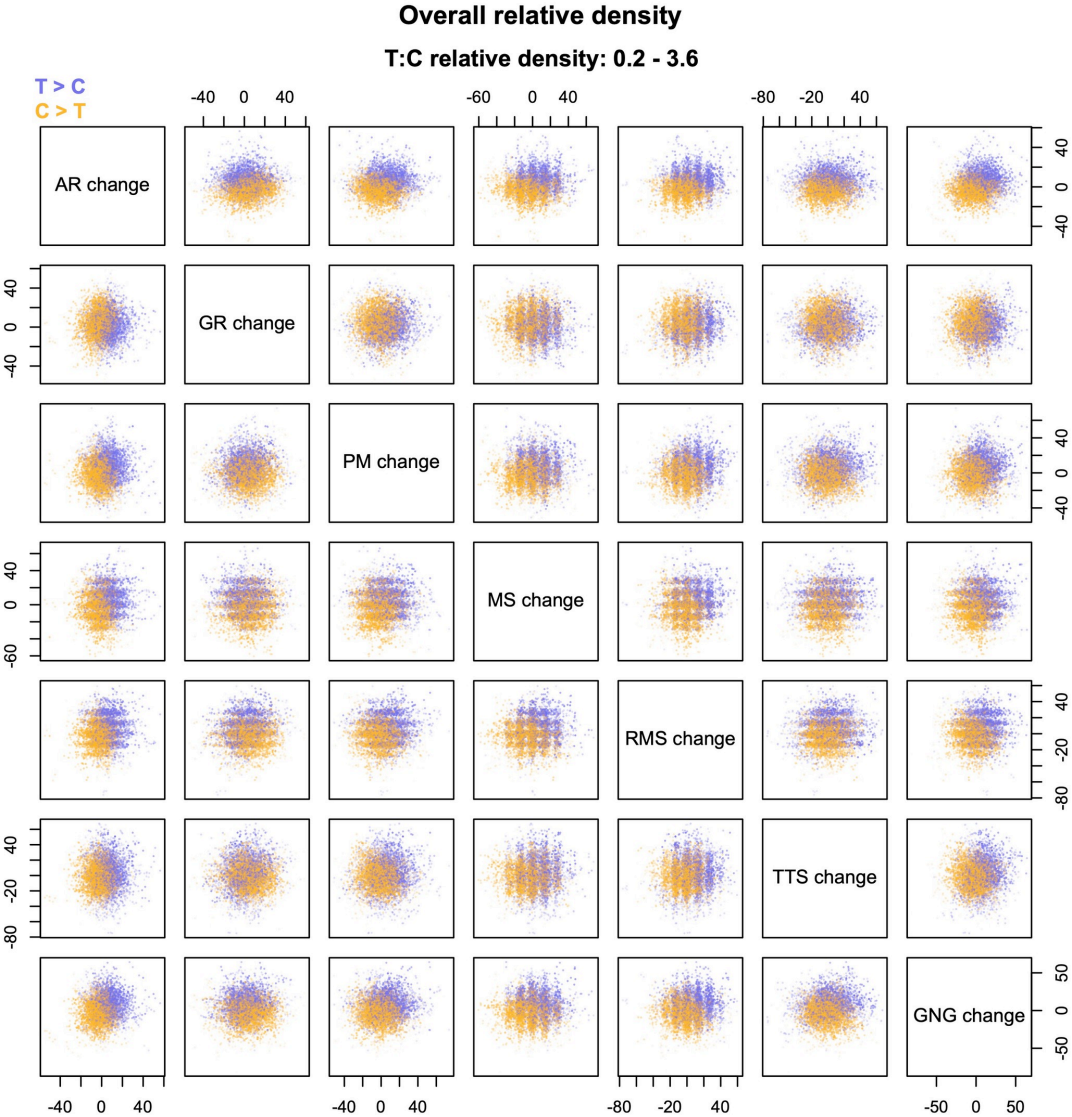
Example of a relative density cloud. The figure compares the performance changes of 2,667 participants who underwent an online cognitive training (T) with that of 2,378 control participants, on a battery of seven neuropsychological tests (data from [11]). Positive scores indicate increased performance between pre- and post-test; negative scores indicate decreased performance. The plots are based on 7,429 probes, with densities estimated from *k* = 52 nearest neighbors. AR = arithmetic reasoning. GR = grammatical reasoning. PM = progressive matrices. MS = memory span. RMS = reverse memory span. TTS = two-target search. GNG = go/no go.

The result is a multivariate *relative density cloud*, which can be explored through its two-dimensional projections in the bivariate scatterplots, as exemplified in Fig 2. In the figure, the gains of participants who underwent a cognitive training are compared with those of a control group on a battery of seven neuropsychological tests (data from [11]; see the legend of Fig 2 for details). Relative density clouds make it possible to identify regions of higher density for the two groups, with darker/more intense shades corresponding to more extreme density ratios. As with univariate plots, relative densities can be interpreted more intuitively as relative frequencies, provided that the two groups being compared are about equally numerous (or can be assumed to be for the purpose of the comparison).

### R function

To make this new visualization tool widely accessible, I provide an easy-to-use R function (*rdclouds*), which is available at https://doi.org/10.6084/m9.figshare.21743537.

This function can be used to plot relative density clouds, both for the overall relative density and for specific components of group differences. The function also draws diagnostic plots that compare the distribution of actual density ratios with a “null” distribution obtained from a random split of the dataset. All the figures in this paper (except Fig 1) were made with *rdclouds* in R 4.1.2 [12]; the source R scripts and data are archived at https://doi.org/10.6084/m9.figshare.21743552.

## Procedure

### Probe generation

The first step of the method is to “probe” the multivariate distribution of the data at a large number of locations. The *rdclouds* function automatically adjusts the number of probes based on the number of variables in the dataset, and hence the larger or smaller size of each scatterplot (from 11,000 probes when there are only two variables to 7,000 with 10 variables). Each probe is a point in *d*-dimensional space, where *d* is the number of variables in the dataset.

The most straightforward way to generate probes is to sample with replacement from the empirical dataset (both groups combined); especially when sample size is comparatively small, coverage can be improved by adding a small amount of multivariate jitter to each point. Jittering helps probe the sparser regions of the distribution more thoroughly, and yields smoother and more readable clouds. At the same time, it tends to “blur” the distribution to some extent, and may partially mask certain features such as floor/ceiling effects, sharp discontinuities, or quantization of the variables. The latter effect is visible in some of the scatterplots of Fig 2, which show vertical and/or horizontal bands when the corresponding variables can only take a small number of discrete values. Without jitter, the fuzzy bands would appear as straight lines, one for each of the possible values. The *rdclouds* function uses a multivariate normal distribution for jitter, with a default standard deviation of 0.2 times the SD of each original variable. Users can change the amount of jitter by specifying a different multiplier or eliminate it altogether if preferred.

### Density estimation

Estimating the density of multivariate data is a computational challenge for standard methods such as kernel density estimation (KDE). With more than a few dimensions, the increase in computational cost makes these methods highly inefficient if not prohibitive. For this reason, most available R packages are limited to one or two dimensions, with a maximum of 6 dimensions in package *ks* [13]. To overcome this limitation, and allow researchers to explore multivariate data in a computationally efficient fashion, relative density clouds rely on *k*-NN density estimates. Specifically, the *rdclouds* function employs a *k-*NN estimator discussed in [14]:

$$\hat{f}\left(x\right)=\frac{1}{NV_{d}}{\left(\frac{\sum_{j=1}^{k}j^{2/d}}{\sum_{j=1}^{k}{∥x_{j}\left(x\right)-x∥}^{2}}\right)}^{d/2}$$ (1)

where *k* is the number of nearest neighbors, *N* is the sample size, *d* is the number of dimensions, *V*_*d*_ is the volume of the *d*-dimensional unit ball, and ‖**x**_*j*_(**x**) − **x**‖ is the Euclidean distance between the point **x** at which density is evaluated and its *j*-th nearest neighbor **x**_*j*_(**x**). Nearest-neighbor distances are obtained with the *RANN* package [15]. In practice, Eq 1 yields density estimates that tend to be somewhat higher in larger samples. For this reason, function *rdclouds* equalizes the group *N*s before density estimation, by sampling (with replacement) from the smaller group the number of cases needed to reach the size of the larger one. To estimate the relative density at each probe, the function simply computes two *k-*NN estimates separately for the two groups and takes their ratio (so that equal densities in the two groups yield a relative density of one). With equal *N*s in the two groups, the formula to calculate the relative density of groups A and B at probe **x** becomes:

$$\frac{{\hat{f}}_{\text{A}}\left(x\right)}{{\hat{f}}_{\text{B}}\left(x\right)}={\left(\frac{\sum_{j=1}^{k}{∥x_{j\text{B}}\left(x\right)-x∥}^{2}}{\sum_{j=1}^{k}{∥x_{j\text{A}}\left(x\right)-x∥}^{2}}\right)}^{d/2}.$$ (2)

Choosing the number of nearest neighbors to consider (*k*) is a problem that lacks a simple, unambiguous solution. Two common rules of thumb found in the applied literature on *k-*NN methods are (a) the square root of the sample size and (b) 1–2% of the sample size [16, 17]. The *rdclouds* function uses the square root of *N* as the default number of neighbors, but users can choose a different rule (1% of *N*) or directly specify their preferred *k*. Especially when sample size is comparatively small, increasing *k* above the suggested value can “smooth out” the estimates, reducing sampling noise and hence yielding cleaner, more readable clouds. However, larger neighborhoods also become less local (particularly when the data are high-dimensional), and excessive values of *k* may end up obscuring local patterns of variation. For a simple illustration, consider the following scenario (from [18]). If data are sampled uniformly from a *d*-dimensional unit hypercube, the edge length of the smallest hypercube that contains the *k*-nearest neighbors of a point is approximately $l\approx {\left(\frac{k}{N}\right)}^{\frac{1}{d}}$. Assuming *k* ≈ *N*/100, the edge of the *k-*NN hypercube is about half as long as that of the unit hypercube when *d* = 7, and about 63% as long when *d* = 10. With *d* = 20, the edge of the *k-*NN hypercube becomes almost 80% as long as that of the unit hypercube. With *d* = 100, the proportion exceeds 95%, meaning that the “neighborhood” of a point covers almost the same distance as the entire sampling space. In general, *k-*NN techniques become less effective as the dimensionality of the data increases [19–22]. Also, larger numbers of variables become progressively harder to visualize in the paired scatterplot format of relative density clouds. For these reasons, the graphical parameters of *rclouds* are optimized for datasets containing up to about 10 variables.

**Fig 3:**
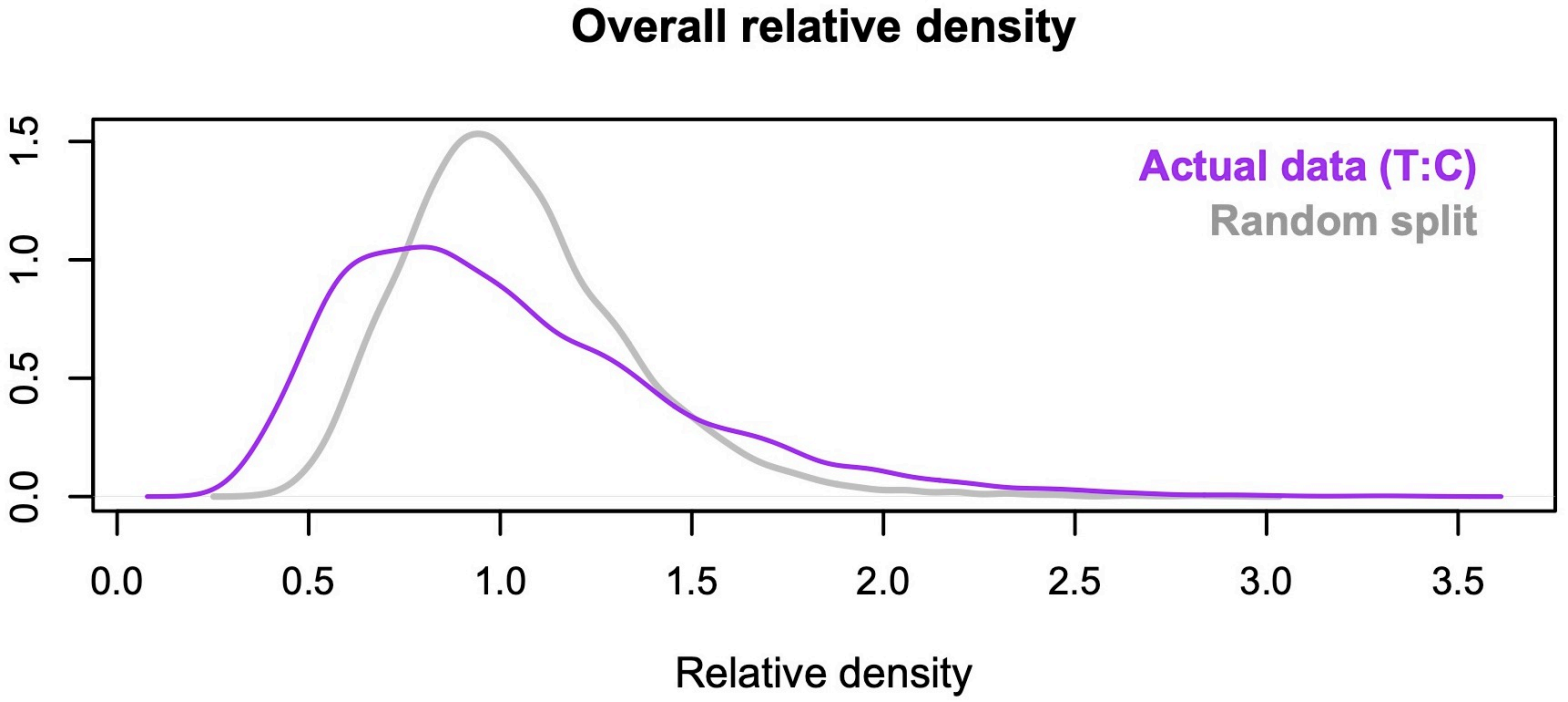
Example of a diagnostic plot. The plot compares the distribution of relative densities in Fig 2 (purple line) with the distribution obtained from a random split of the data, with equal numbers of cases from the two groups in each randomized half (gray line).

Even if the two groups being compared come from the same distribution, local relative densities can fluctuate away from one owing to sampling error and noise in the *k-*NN estimates, especially in low-density regions of the distributions. As I explain below, relative density clouds use transparency to reduce the visual influence of ratios close to one, and emphasize more extreme ratios that are less likely to arise by chance. Still, it can be useful to perform a diagnostic check by comparing the distribution of estimated relative densities with a “null” distribution produced only by stochastic processes. By default, the *rclouds* function draws diagnostic plots like the one shown in Fig 3. The purple line shows the actual distribution of density ratios (in this case, the distribution for the clouds displayed in Fig 2). The gray line shows the distribution obtained from a random split of the data, with equal numbers of cases from the two groups in each randomized half. In Fig 3, the distributions are fairly distinct, and relative density values from the actual data clearly predominate at both tails. In contrast, a close correspondence between the two lines would indicate a genuine lack of differences between the groups, a large amount of noise in the estimates, or both. In the latter case, even the highest and lowest density estimates are likely to reflect sampling error instead of true group differences. This problem can usually be ameliorated by increasing the value of *k*, especially when sample size is comparatively small (see above).

### Plotting

Once relative densities are estimated, the probes are plotted at their respective coordinates, with different colors to indicate whether density is higher in one or the other group, and transparency inversely related to the extremity of the ratio. In *rdclouds*, the alpha value of each point is inversely proportional to the logarithm of the corresponding density ratio, so that a density ratio of one corresponds to alpha = 0 (maximum transparency) and the most extreme observed ratio—in either direction—corresponds to alpha = 1 (maximum opacity). The most extreme ratio can be identified *globally* (across all the plots) or *locally* (within each plot). Using the global maximum as a reference makes it easier to visually compare the relative strength of different effects within a given decomposition sequence (see below). The logarithmic scaling of transparency acts as a “compression filter” that limits the potential influence of isolated outliers, further contributing to reduce noise in the plots.

The resulting scatterplots are two-dimensional projections of a multivariate cloud of relative density estimates. They convey two kinds of information: (a) background information about the absolute density of the distribution (from the spatial distribution of probes), and (b) information about the relative density of the two groups (from the color/shade of probes). Decoding this information is made easier by judicious graphical choices about the colors used for plotting, the size/number of the points in each plot, and so forth. To further assist interpretation, the plots generated by *rdclouds* include the numeric range of the relative densities that are displayed graphically in the clouds (see e.g., Fig 2).

## Effect decompositions

One of the most attractive features of relative distribution methods [5] is the ability to decompose the overall pattern of group differences into multiple components that reflect the independent effects of differences in location, scale, shape, and so forth. Relative density clouds can be used to implement a graphical version of this technique, by transforming the data in suitable ways before generating probes and estimating densities. Specifically, function *rdclouds* offers three decomposition sequences, labeled LS (*location*, *shape*), LSS (*location*, *scale*, *shape*), and LSCS (*location*, *scale*, *covariation*, *shape*).

**Fig 4:**
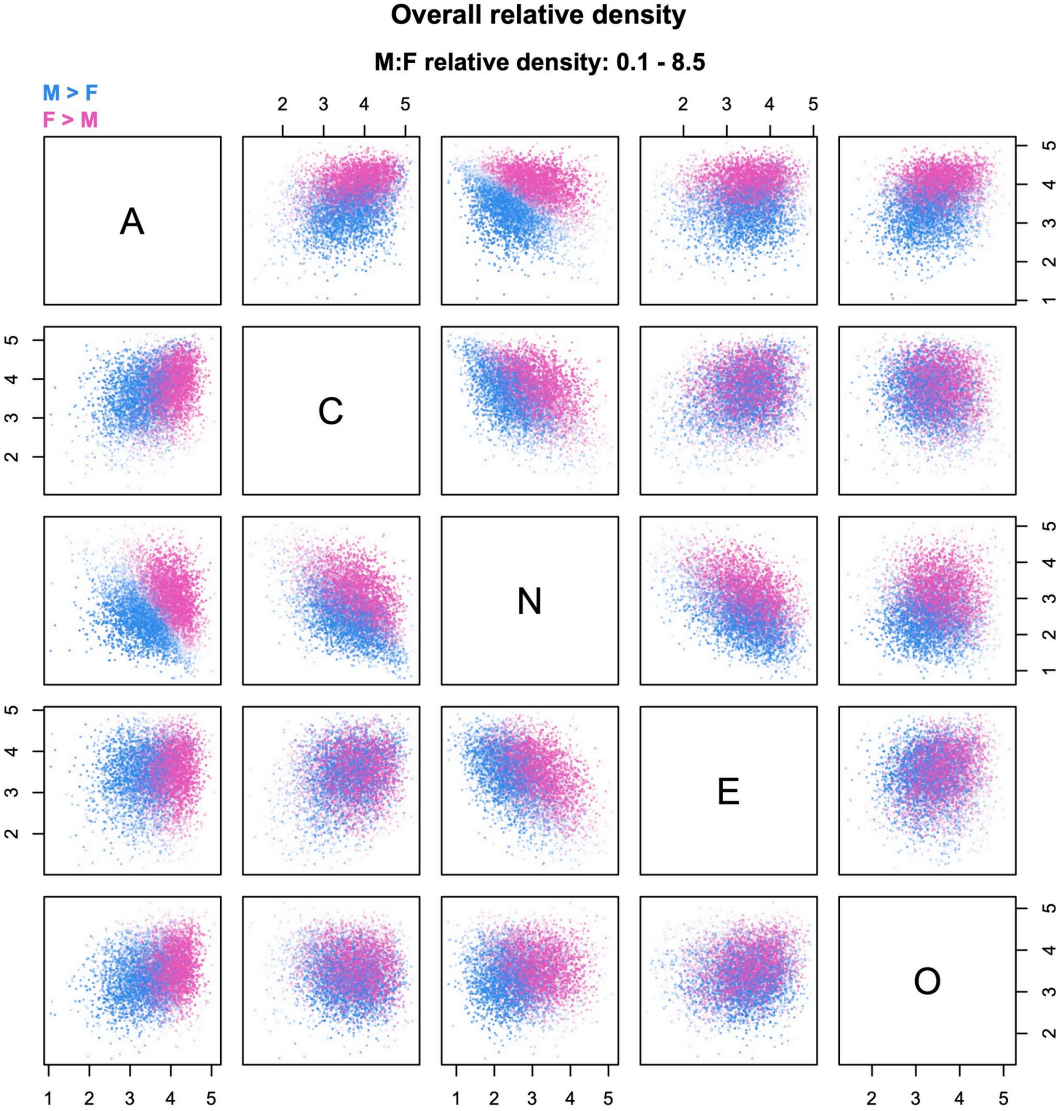
Relative density cloud of sex differences in personality. The figure compares the male (M) and female (F) distributions of the Big Five personality traits (total *N* = 100,000, 50% females). The plots are based on 8,000 probes, with densities estimated from *k* = 224 nearest neighbors. A = Agreeableness. C = Conscientiousness. N = Neuroticism (emotional instability). E = Extraversion. O = Openness to experience.

**Fig 5:**
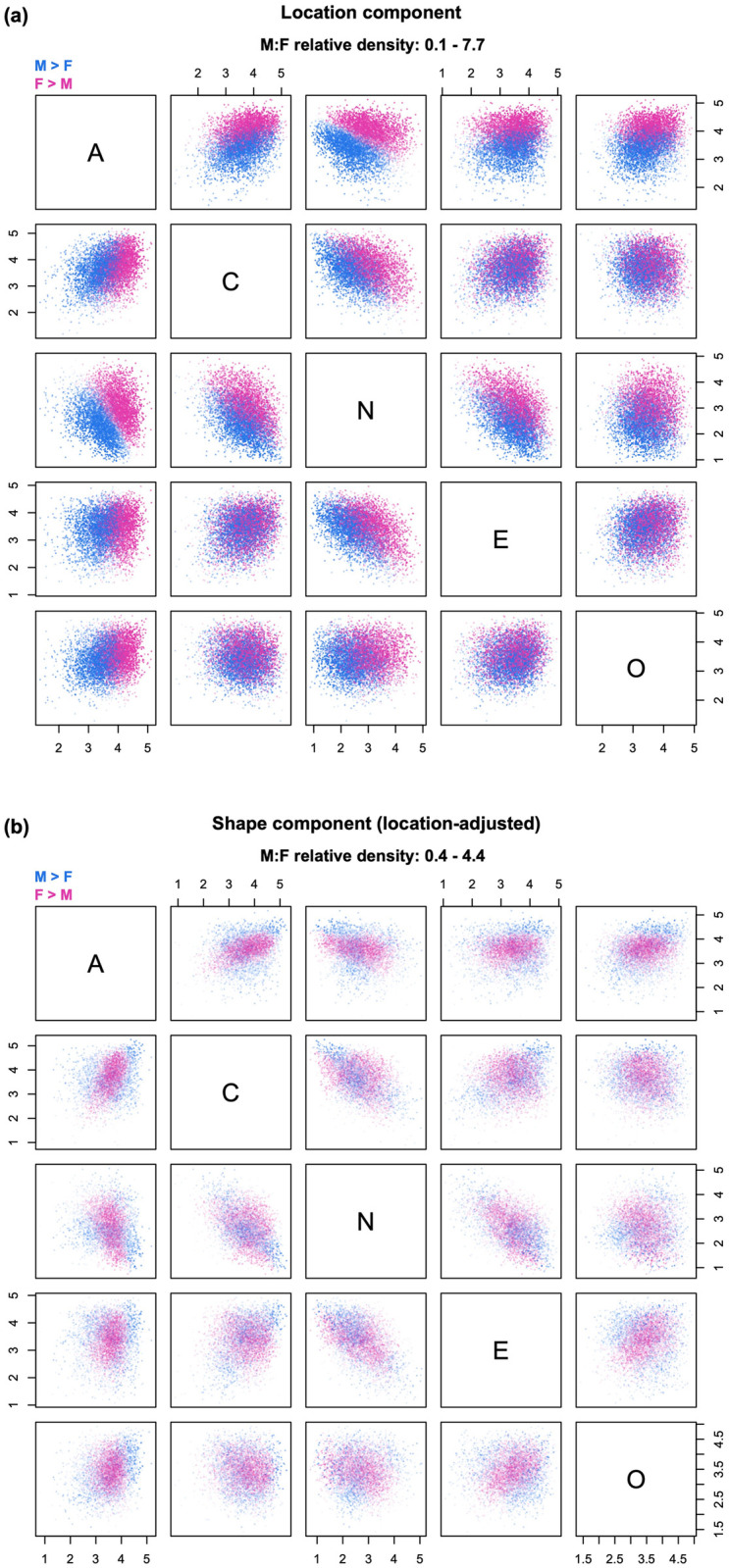
Example of decomposition into location and shape. The relative density clouds show the decomposition of the overall relative densities in Fig 4 into (a) a location component and (b) and shape component adjusted for sex differences in centroids. Note: transparency is referenced to the global maximum across Figs 4–7.

**Fig 6:**
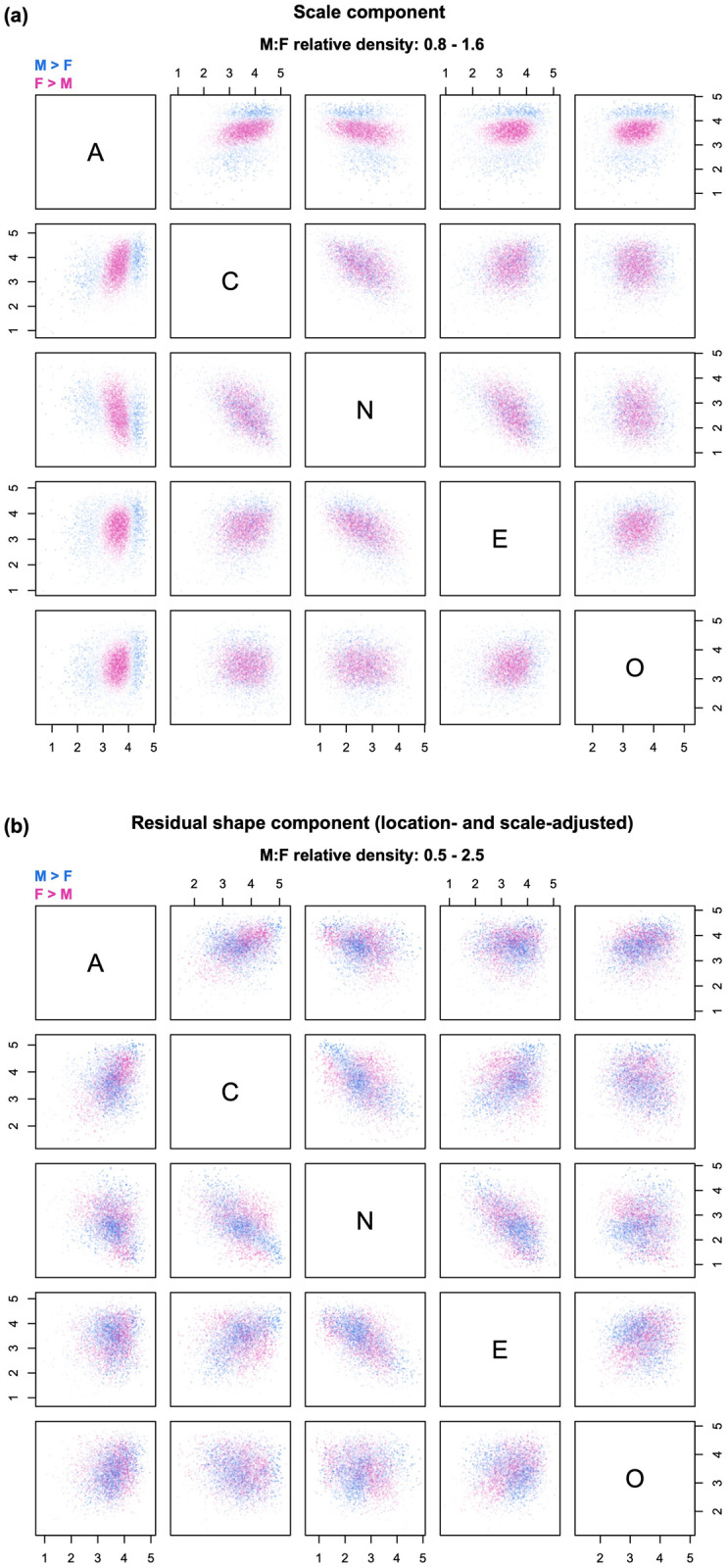
Example of decomposition into scale and residual shape. The relative density clouds show the decomposition of the shape component of Fig 5b into (a) a scale component and (b) a residual shape component adjusted for sex differences in centroids and variances. Note: transparency is referenced to the global maximum across Figs 4–7.

**Fig 7:**
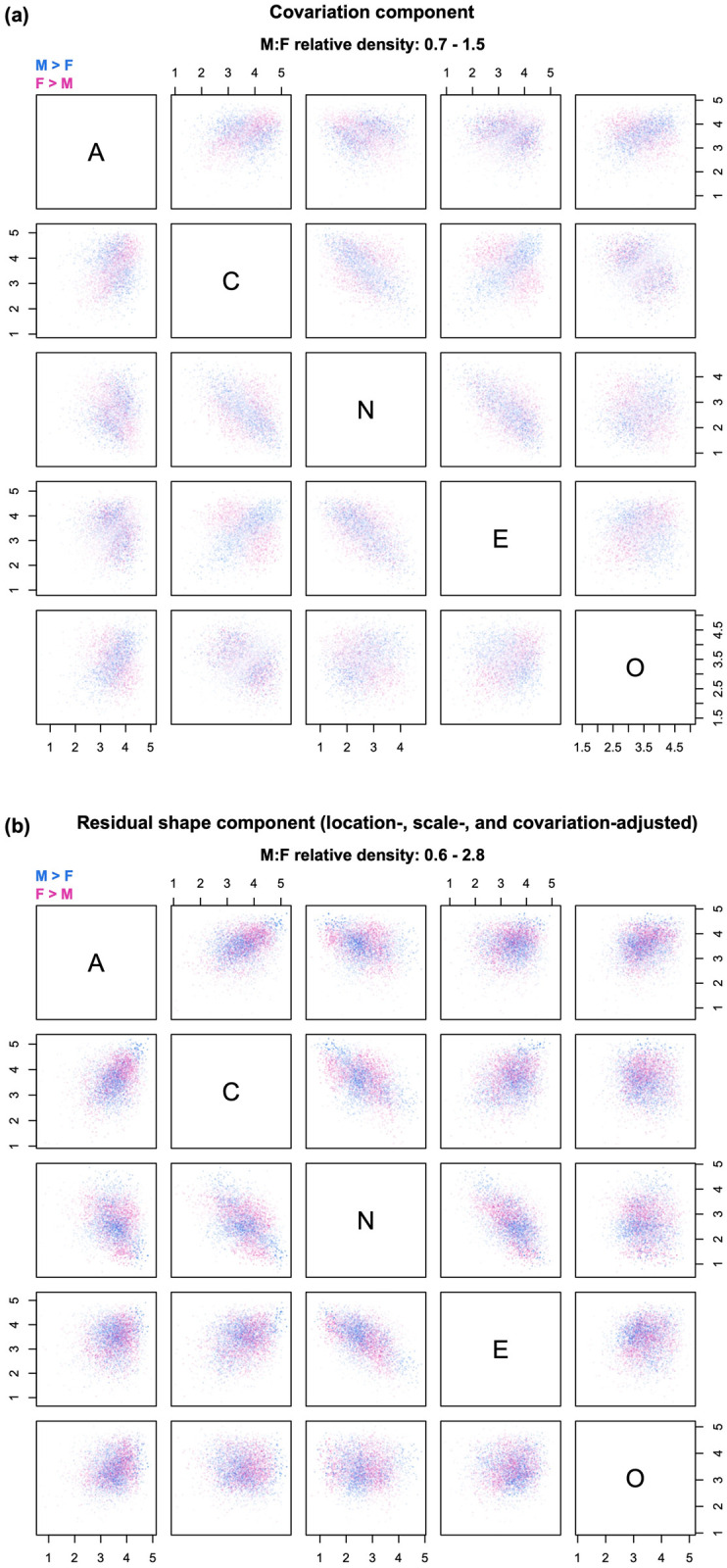
Example of decomposition into covariation and residual shape. The relative density clouds show the decomposition of the residual shape component of Fig 6b into (a) a covariation component and (b) a residual shape component adjusted for sex differences in centroids, variances, and correlations. Note: transparency is referenced to the global maximum across Figs 4–7.

In Figs 4 to 7, I demonstrate these decompositions with a dataset of sex differences in Big Five personality traits—Agreeableness, Conscientiousness, Neuroticism, Extraversion, and Openness—for a sample of 100,000 participants from the United States (equal numbers of men and women), randomly selected from a larger dataset described in [9]. Fig 4 shows the overall relative density; women are the reference group, whereas men serve as the comparison group. The plots identify two large regions in multivariate space where men and women are over-represented, suggesting a strong effect of mean differences (i.e., location shifts). Sex differences are especially clear-cut along the axis that goes from high to low levels of Agreeableness and Neuroticism (first row/third column or third row/first column in Fig 4). From this plot alone, it is hard to identify additional effects besides that of differences in location; this is where decomposition sequences can make a key contribution and significantly deepen one’s understanding of the data. Note that transparency in Figs 4–7 is referenced to the global maximum across the plots, to permit a quick visual assessment of the relative strength of different components.

### LS decomposition

The first two sequences available in *rdclouds* replicate the basic decomposition procedure described in [4, 5]. In the LS sequence (Fig 5), the effect of location shift (i.e., the location component) is computed as the density ratio between two distributions: a transformed version of the reference distribution (group B) that has been mean-shifted to have the same centroid as the comparison group A, and the untransformed distribution of group B (Fig 5a; for details see [5]). Then, the shape component is found as the density ratio between the original distribution of group A and the mean-shifted distribution of group B (Fig 5b). The shape component answers the counterfactual question “what would group differences look like if both groups had the same centroid?”

As expected, the location component displayed in Fig 5a is substantial, and closely replicates the overall pattern of sex differences seen in Fig 4. However, the shape component in Fig 5b reveals the existence of other effects, including the possibility of scale shifts due to somewhat greater male variability in most of the traits [1, 3]. After equalizing the centroids, men become over-represented in certain extreme regions; for example, there is an identifiable cluster of probes with male-biased density (dark blue points) at high levels of Agreeableness, Extraversion, Conscientiousness, higher-than-average levels of Openness, and low levels of Neuroticism. Women are generally over-represented in the central part of the distribution, but the female-dominated region extends to the low end of Neuroticism.

### LSS decomposition

The LSS sequence goes one step further, and decomposes the shape component displayed in Fig 5b into a scale component and a residual shape component adjusted for both location and scale (Fig 6). Specifically, the effect of scale shifts is computed as the ratio between a transformed version of the distribution of group B that has been mean-shifted and rescaled to have the same centroid and variances as group A, and its mean-shifted version described in the context of the LS sequence (Fig 6a). Then, the residual shape component (adjusted for both location and scale) is found as the density ratio between the original distribution of group A and the mean-shifted, rescaled distribution of group B (Fig 6b). The corresponding question is, “what would group differences look like if the groups had the same centroid *and* variances?” The LSS decomposition helps separate the specific contribution of differences in scale from that of other features such as skewness, kurtosis, etc. It is particularly useful when there are reasons to anticipate the existence of systematic differences in variability between the two groups (as is often the case in comparisons between the sexes [1, 3]).

Inspection of Fig 6a confirms a pattern of somewhat higher variability in men, most pronounced along the dimension of Agreeableness (first row and first column). Blue “halos” at the outer edge of the distribution are also visible along the dimensions of Extraversion and Openness. Of note, scale differences in Agreeableness are likely exaggerated by a marked ceiling effect on questionnaire scores (clearly visible in Fig 4), which constrains female variation in the upper range of the trait (more on this below). The region of higher female density reaches both the high and low end of Neuroticism, but with values of Agreeableness, Extraversion and Openness that remain closer to the distribution mean. Having accounted for differences in scale, the residual shape component in Fig 6b is less patterned than that in Fig 5b; there are still indications of male and female over-representation in certain regions, even net of location and scale effects. For example, men are over-represented in the region with the highest levels of Neuroticism, perhaps because their distribution has a thicker tail in that direction.

### LSCS decomposition

In multivariate contexts, there is another recurrent source of group differences besides location and scale—namely, differences in patterns of covariation among the variables. Changes in correlational structure can produce relative density changes—and hence differences in “residual shape”—even in the limit case of two multivariate normal distributions with equal centroids and variances (i.e., no contribution from higher moments). The LSCS sequence extends the approach presented in [5] to isolate the effect of group differences in covariation, by decomposing the residual shape component of the LSS sequence into a *covariation component* and a location-, scale-, and covariation-adjusted residual shape component (Fig 7). This is accomplished by further transforming the mean-shifted, rescaled distribution of group B so that it has the same correlation matrix as group A. Because the distribution also has the same variances as group A, this corresponds to equalizing the covariance matrices of the two distributions.

More formally, the goal is to transform the centered *N*_*B*_ × *d* data matrix **X**_B_ into a matrix $X_{\text{B}}^{*}$ that has the same covariance matrix as *N*_*A*_ × *d* data matrix **X**_A_. The first step is to *whiten* **X**_B_ with an appropriate whitening matrix **W**_B_. Function *rdclouds* employs the *ZCA-cor* whitening transformation, which maximizes correlations between the original and transformed variables [23] (performed with package *whitening* [24]). The ZCA-cor whitening matrix for **X**_B_ is

$$W_{\text{B}}=\;R_{\text{B}}^{-\frac{1}{2}}V_{\text{B}}^{-\frac{1}{2}},$$ (3)

where **R** is a correlation matrix and **V** is a diagonal matrix of variances. Whitening **X**_B_ yields a whitened data matrix **Z**_B_, in which the variables are all orthogonal with unit variance:

$$Z_{\text{B}}={\left(W_{\text{B}}X_{\text{B}}^{\text{T}}\right)}^{\text{T}}.$$ (4)

The inverse of a whitening matrix is known as a *coloring* matrix. The ZCA-cor coloring matrix for **X**_A_ is $W_{\text{A}}^{-1}=\;V_{\text{A}}^{1/2}R_{\text{A}}^{1/2}$; this matrix can be used to transform **Z**_B_ into $X_{\text{B}}^{*}$, as follows:

$$X_{\text{B}}^{*}\;={\left(W_{\text{A}}^{-1}Z_{\text{B}}^{\text{T}}\right)}^{\text{T}}.$$ (5)

Matrix $X_{\text{B}}^{*}$ can now be mean-shifted so that it also has the same centroid as **X**_A_.

The covariation component of the relative density can now be computed as the ratio between the mean-shifted, rescaled, and colored distribution of group B and its mean-shifted and rescaled version (Fig 7a). Then, the new residual shape component (adjusted for location, scale, and covariation) is found as the density ratio between the original distribution of group A and the mean-shifted, rescaled, and colored distribution of group B (Fig 7b). The residual shape component of the LSCS sequence answers the question, “what would group differences look like if the groups had the same centroid, variances, and correlation matrix?” The LSCS decomposition can be especially useful when there are reasons to suspect the existence of meaningful group differences in correlational structure.

**Fig 8:**
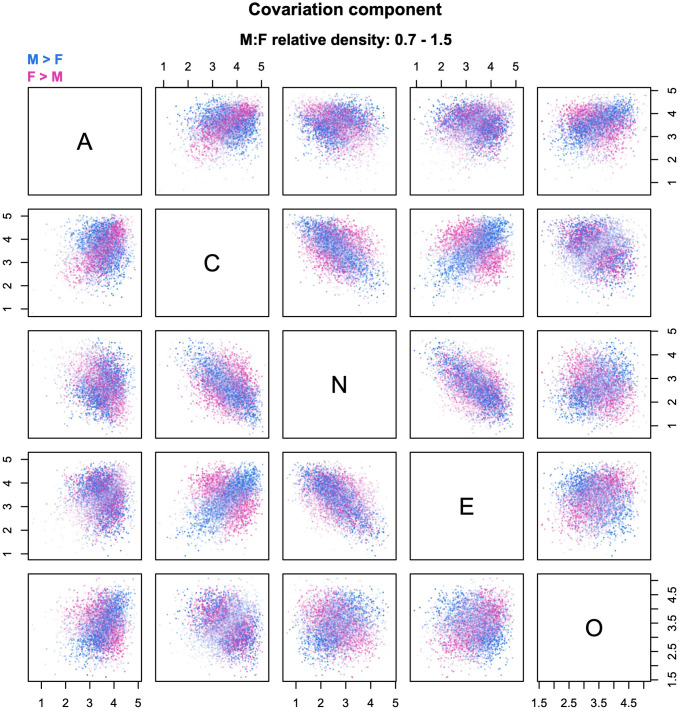
Example of transparency referenced to the local maximum. This is the same relative density cloud of Fig 7a (covariation component of sex differences in Big Five personality traits), with transparency referenced to the local maximum to improve the legibility of the plots.

In the personality example, the effect of covariation shifts is rather weak, as is apparent from the plots in Fig 7a. This is consistent with the finding that, as a rule, personality traits show approximately the same correlations in the two sexes [1, 25]. Accordingly, the residual shape component in Fig 7b is very similar to that in Fig 6b, and can be assumed to mainly reflect group differences in skewness, kurtosis, and/or higher moments of the distributions. If one wishes to examine the covariation component in more detail despite its comparatively small contribution to sex differences, it is sufficient to plot the corresponding clouds with transparency referenced to the local maximum, as shown in Fig 8.

The default decomposition sequences in *rclouds* include only one shape component as the last step in the sequence, following the approach outlined in [5]. However, users can also plot “augmented” sequences (labeled LSS+ and LSCS+) that include all the steps of the higher-level decompositions. Hence, the LSS+ sequence yields five plots (overall relative density plus location, shape, scale, and residual shape components); whereas the LSCS+ sequence produces all the seven plots illustrated in Figs 4–7.

## Some notes on interpretation

Relative density clouds are a flexible visualization tool for the study of group differences. Just like other powerful exploratory methods, they require careful interpretation; researchers must be aware of potential pitfalls to avoid drawing incorrect or unwarranted inferences.

### Multivariate vs. bivariate densities

The first thing to keep in mind is that relative density clouds are multivariate objects; each probe is a *d*-dimensional point that appears—with the same color and transparency—in all the two-dimensional projections within a plot (i.e., the bivariate scatterplots). Stated differently, bivariate scatterplots show two-dimensional projections of a single cloud of *multivariate* relative densities calculated simultaneously over the entire set of variables, not *bivariate* relative densities calculated on two variables at a time. If one intends to visualize bivariate densities, one must run the procedure on subsets of two variables each.

**Fig 9:**
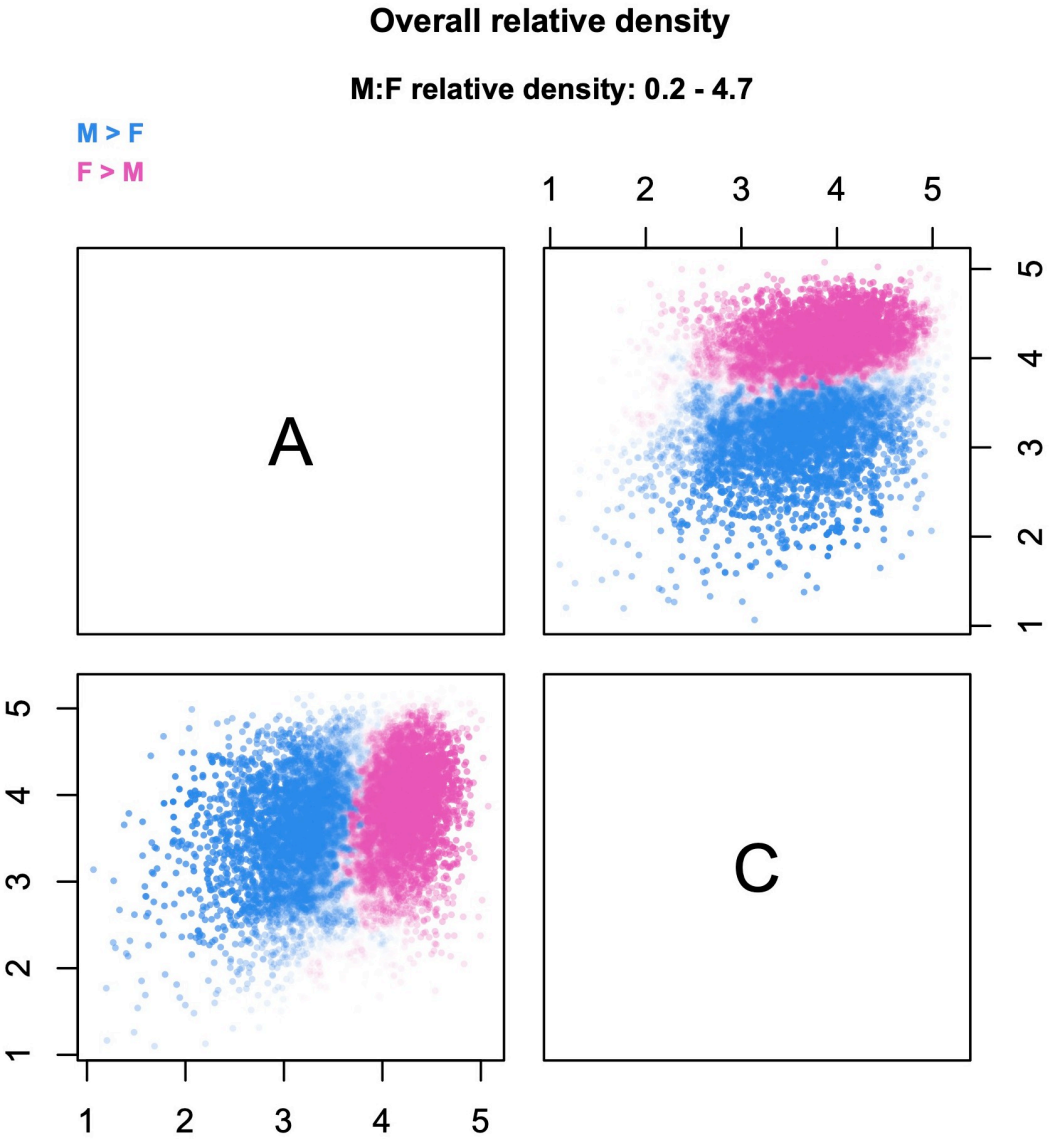
Example of bivariate relative densities. The figure shows the relative density cloud for sex differences in traits Agreeableness (A) and Conscientiousness (C), plotted from the same dataset used for Figs 4–8. The plots are based on 11,000 probes, with densities estimated from *k* = 224 nearest neighbors.

To illustrate, Fig 9 shows the bivariate relative density for Agreeableness and Conscientiousness. This plot corresponds to the upper left corner of Fig 4. While the general pattern is similar, there are some important differences. Most notably, the male- and female-dominated regions of the distribution in Fig 9 are separated by a borderline region with high transparency (i.e., relative densities close to one); whereas in Fig 4 the pink and blue regions overlap, yielding a purple area. This is because the probes shown in Fig 4 are located in a five-dimensional space; when they are projected on a two-dimensional plane, regions of higher male and female density can overlap one another, even though they are separated along other dimensions of the multivariate space. For example, in Fig 4, the Agreeableness-Neuroticism scatterplot (first row/third column or third row/first column) shows that, where the two regions have the same level of Agreeableness, they differ with respect to Neuroticism. This pattern yields two cleanly separated regions on the Agreeableness-Neuroticism plane, but overlapping regions when the cloud is projected on the Agreeableness-Conscientiousness plane (first row/second column or second row/first column).

### Regions in multivariate space

The example discussed in the previous section illustrates the more general point that individual scatterplots should not be interpreted in isolation. When there are more than three variables, the shape of the *d*-dimensional cloud in cannot be visualized in its entirety; but it can be inferred—in partial “chunks”—by triangulating among multiple two-dimensional views of the probes. This point applies in full when one is trying to locate and characterize a specific region of the distribution. Sometimes, there are clusters of probes that remain clearly identifiable throughout multiple plots, as for example the dark blue probes with high Agreeableness, Conscientiousness, Extraversion, and low Neuroticism in Fig 5b (also visible in Figs 6b and 7b). Other times, one lacks such distinctive landmarks; the two-dimensional projections are compatible with multiple arrangements of probes and must be interpreted accordingly, using multiple viewpoints to check one’s interpretation. In many cases, relative density clouds are going to be *compatible* with certain patterns in the data, rather than dispositive; investigators should then use other sources of information (e.g., targeted analyses of the group data) to substantiate their exploratory findings.

**Fig 10:**
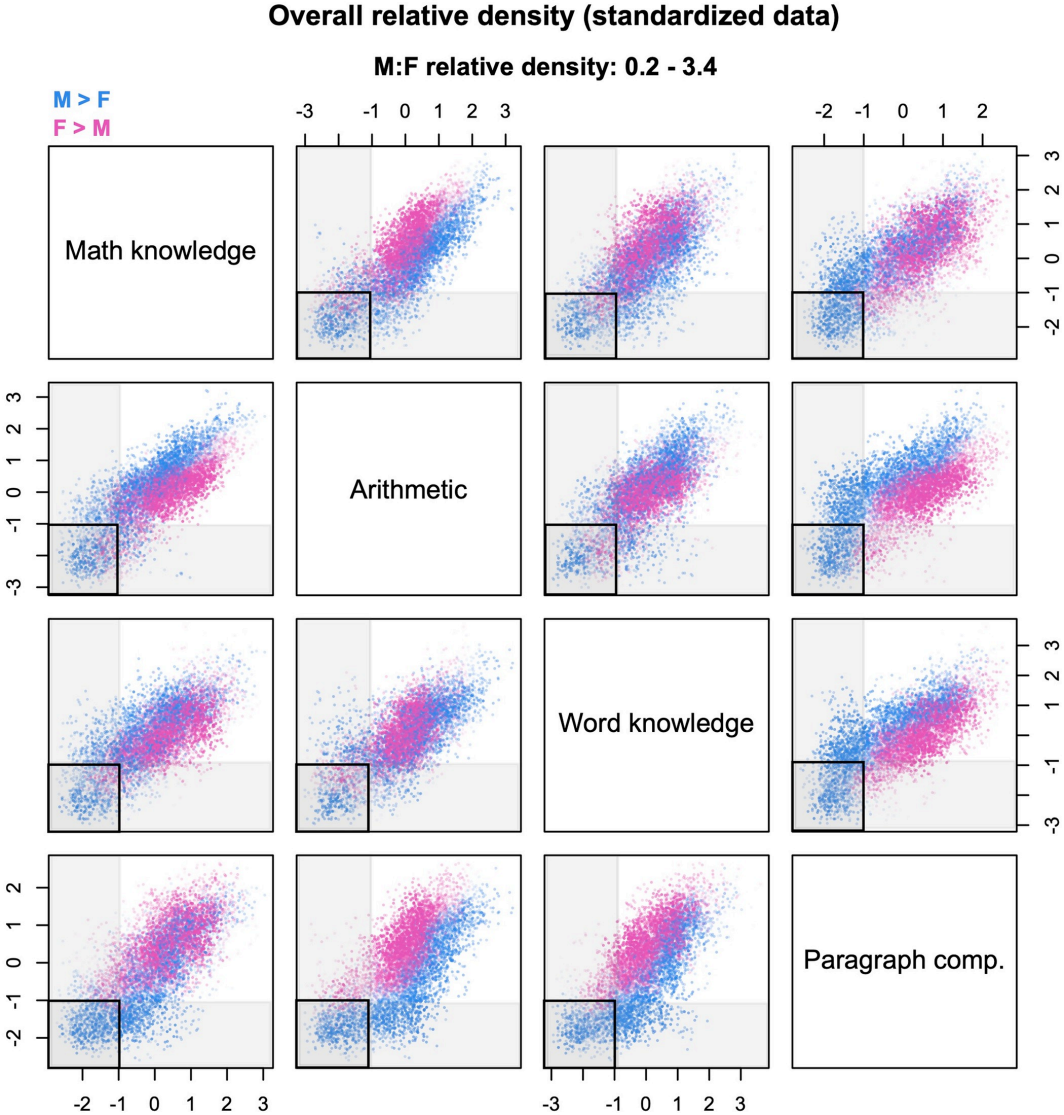
Multivariate interpretation of regions in the cloud. The cloud compares the male (M) and female (F) distributions of cognitive performance on four tasks of the ASVAB battery. The data are from the 1997 National Longitudinal Survey of Youth and comprise 3,582 males and 3,494 females (complete cases only). The plots are based on 8,500 probes, with densities estimated from *k* = 60 nearest neighbors. Black rectangles in the scatterplots identify regions with scores < 1 SD below the mean on both tasks. Gray bands identify regions with scores < 1 SD below the mean on a single task.

For a different perspective on the same problem, consider Fig 10. This figure is based on data from the 1997 National Longitudinal Survey of Youth (NLSY97); the relative density cloud compares males and females on four cognitive tests—two verbal, two mathematical—of the Armed Services Vocational Aptitude Battery (ASVAB). The scores have been standardized with respect to the overall mean and variance. Suppose that investigators are interested in the sex distribution of youth who perform poorly across the board, operationalized as scoring less than one standard deviation below the mean on all the four tests. This criterion identifies a four-dimensional hyperrectangle in the multivariate space; in Fig 10, its projections are shown as black rectangles. Even a cursory inspection of the figure reveals that the rectangles do not capture exactly the same probes across different scatterplots. Notably, some rectangles contain almost only blue probes (male-biased densities), whereas others contain a significant fraction of pink probes (female-biased densities). To convey a better sense of why this happens, the figure also shows gray bands that identify scores of less than 1 SD on each individual test. In each scatterplot, the gray bands highlight sets of probes that are excluded from the target rectangle but could potentially end up within the rectangle in other scatterplots. Specifically, probes that fall within the horizontal band might be captured by the rectangles in scatterplots that lie on the same row; while probes that fall within the vertical band might be captured by the rectangles in scatterplots that lie on the same column. Only some of the probes—if any—are captured by *all* the rectangles and hence satisfy the investigators’ criterion of “low scores across the board”.

A closer inspection of Fig 10 shows that the low-performance rectangle for math knowledge and paragraph comprehension (first row/fourth column or fourth row/first column) contains almost no female-biased probes. This is a crucial bit of information, because any probe that appears only in *some* of the projections of the hyperrectangle—like the vast majority of the female-biased probes—does not actually lie within the hyperrectangle itself. (Conversely, any probe that *does* lie withing the hyperrectangle must appear in all of its projections.) Hence, Fig 10 indicates that boys are consistently over-represented among youth who perform poorly across the board.

### High-dimensional phenomena

Whenever one is dealing with more than a handful of dimensions, it is important to remember that our geometric intuitions—which are based on the experience of a three-dimensional world—can become seriously misleading when they are applied to high-dimensional spaces. For an illustration of the counter-intuitive phenomena that take place in high dimensions, consider a normal distribution of points. In the familiar uni- and bivariate cases, the mass of the distribution clusters around the mean/centroid, and only a small proportion of points are located in the tails. But as dimensionality increases, a larger proportion of the probability mass becomes concentrated in the *tail* region, where density is comparatively low. That is, the majority of the points move far away from the centroid, along a progressively thinner “shell” that envelopes a mostly empty interior [26]. While the points disperse further in space, the distribution of the distances between them gets proportionally narrower; as more and more dimensions are added, all the points in the distribution tend to become approximately equally distant from one another, as well as from the centroid. This phenomenon is known as *distance concentration* and contributes to limit the usefulness of *k*-NN methods in high-dimensional problems [19, 26, 27]. The effects I just described are strongest when the variables are orthogonal to one another, so that the *effective dimensionality* of the dataset equals the number of variables. As correlations among variables increase, the effective dimensionality shrinks, with the result that (Euclidean) distances grow less steeply and concentrate at a slower pace [20, 28].

High-dimensional phenomena are not merely statistical curiosities, and affect multivariate data analysis in all sorts of ways [26, 27]. This includes the visualization of multivariate distributions, which is necessarily filtered through low-dimensional projections (for in-depth discussion see [29]). For the purpose of the present paper, the key take-home point is the following: when interpreting relative density clouds, it is important to remember that what looks like a concentration of probes at the center of the distribution might actually be the two-dimensional projection of a mostly empty “shell” in multivariate space. Fortunately, this problem becomes especially severe when the data span more than about 10–20 dimensions (see Giraud, 2015), which is probably beyond the point at which relative density clouds become too visually crowded to be practical. With less than about ten variables, the problem tends to remain at manageable levels, especially if the variables are mutually correlated. For example, the Big Five dataset that comprises the sample displayed in Figs 4–9 has between four and five effective dimensions, and the average distance between each data point and the centroid amounts to approximately two multivariate SDs (details in Del Giudice, 2021b). These values are far from extreme, supporting a reasonably “intuitive” interpretation of the plots in Figs 4–9. However, one should become more careful as the number of variables increases; an upper limit of ~10 variables may be used as a sensible rule of thumb.

### Measurement artifacts

The issue of measurement artifacts is not unique to relative density clouds, and is just as relevant to the interpretation of univariate relative density plots such as the one in Fig 1. Systematic forms of measurement error distort the distribution of the original variables, for example by constraining their high and/or low values (ceiling/floor effects), adding larger amounts of noise to certain parts of the distribution, and so forth. In turn, these distorting effects may give rise to artifacts in patterns of location, scale, covariation, and/or other aspects of distribution shape. Of course, relative density clouds will display existing group differences regardless of their sources—whether they represent true effects, measurement artifacts, or a mixture of both. Investigators should always be mindful of the possible presence of measurement artifacts and their potential impact on patterns of relative density.

Earlier in the paper, I noted that Agreeableness scores show a marked ceiling effect, which likely contributes to inflate the apparent size of sex differences in scale (see Figs 4–6). Naturally, floor/ceiling effects can change other moments of the distributions besides variance (e.g., skewness). The supporting information (S1 File) illustrates the impact of ceiling effects using a simulated dataset. In general, simulations can be extremely useful to clarify the effects of specific kinds of measurement errors, and visualize combinations of multiple artifacts in the same data.

## Conclusion

The study of multivariate group differences presents a host of challenges, but also many opportunities for methodological innovation. Relative density methods offer a powerful, flexible toolkit for the analysis of univariate differences (Handcock & Morris, 1998, 1999). In this paper I introduced a novel visualization method that brings some of the same advantages to fruition in the context of multivariate research. Relative density clouds can assist in the exploration of complex patterns of group differences, and help break them down into simpler, more interpretable effects. They should prove useful to researchers in disparate fields who are looking for new sources of insight into their data.

## Supporting information

S1 File Illustration of the impact of ceiling effects. (PDF)
