# Supplementary material for: Relative density clouds: Visualizing and exploring multivariate patterns of group differences
Source: PLoS One. 2023 Jun 27;18(6):e0287784. doi: 10.1371/journal.pone.0287784 (PMC10298761; doi:10.1371/journal.pone.0287784)
Supplement: S1 File — (PDF) [file pone.0287784.s001.pdf]

Supplementary material for

Relative Density Clouds:
Visualizing and Exploring Multivariate Patterns of Group Differences

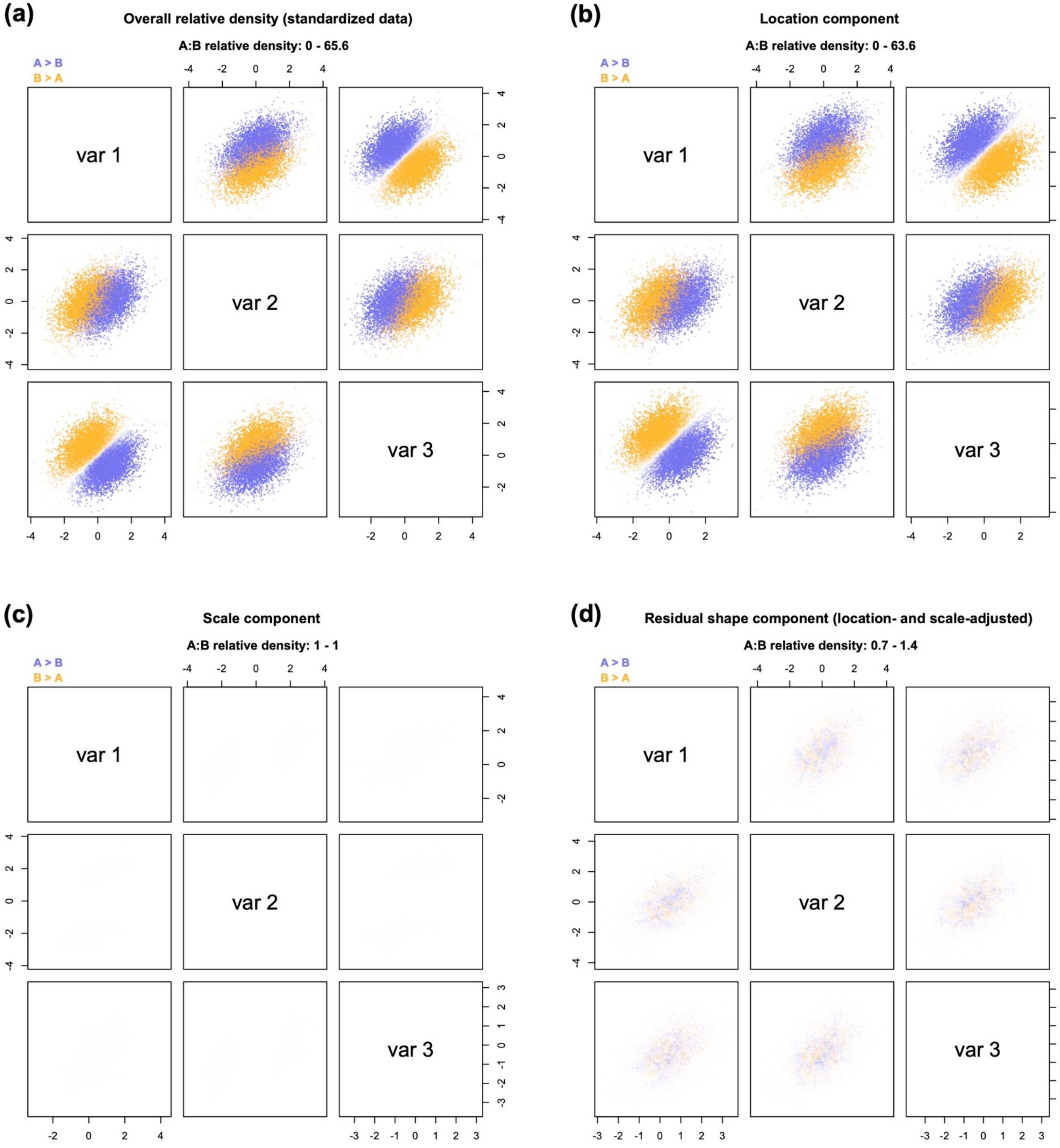

Fig. S1. Relative density clouds (LSS decomposition) for two groups of N = 50,000 each. The data come from two multivariate normal populations with the same covariance matrix, but different means on variables 1 and 3. Each variable is calculated as the sum of five normally distributed items. As expected, the scale component in panel (c) is effectively nonexistent, and the residual shape component in panel (d) (which in this case captures only sampling error) is weak and unstructured.

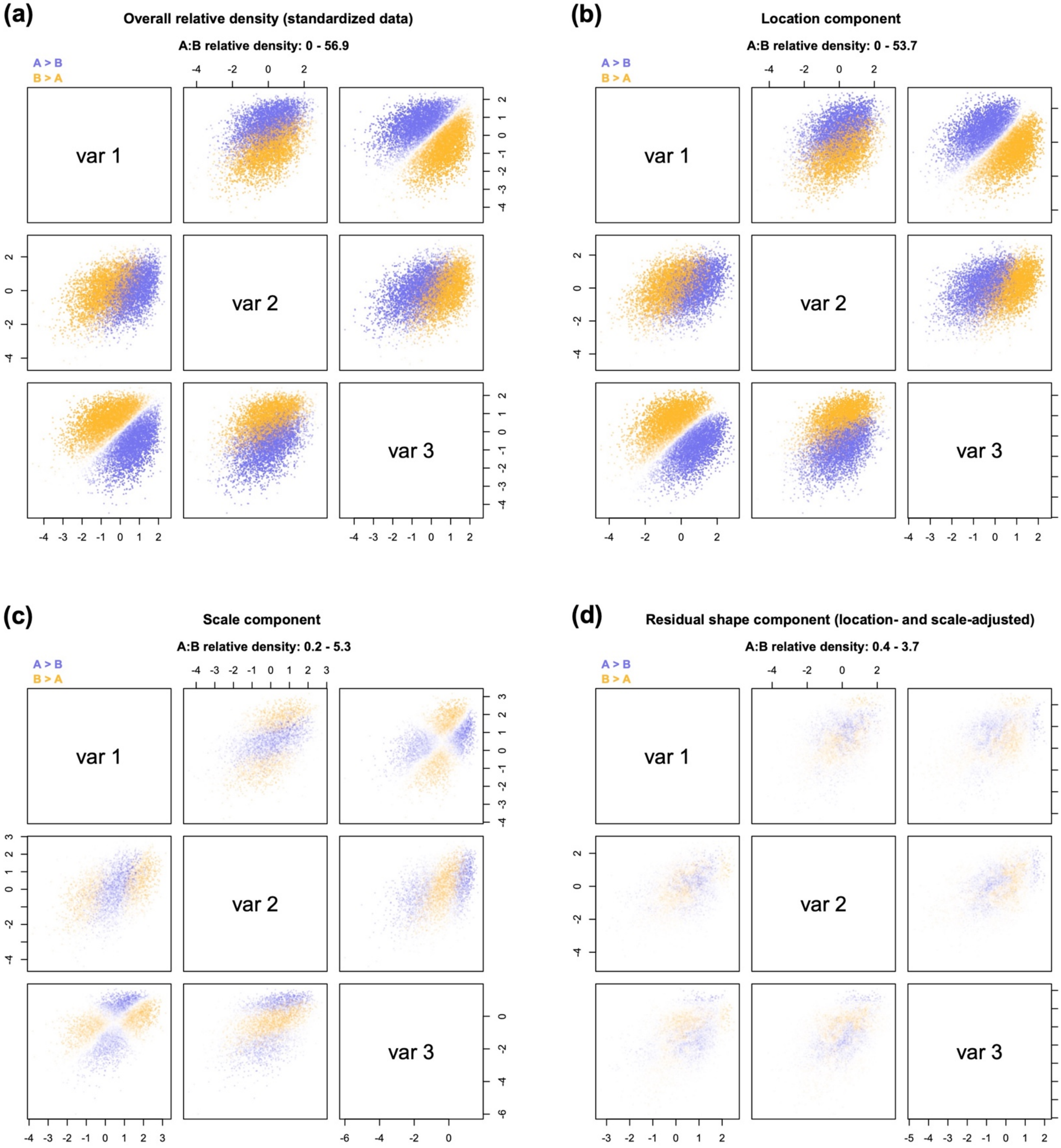

Fig. S2. Relative density clouds (LSS decomposition) for the same groups of Figure S1, after introducing a ceiling effect that constrains the upper range of the variables. The effect is visible in panel (a), and is obtained by placing a threshold on the score of individual items before summing them to yield the corresponding variables. This ceiling effect gives rise to the artifactual scale component of panel (c), as well as the somewhat stronger and more patterned residual shape component shown in panel (d) (compare with Fig. S1d).
